# *Anaplasma phagocytophilum* Antibodies in Humans, Japan, 2010–2011

**DOI:** 10.3201/eid2003.131337

**Published:** 2014-03

**Authors:** Gaowa, Yuko Yoshikawa, Norio Ohashi, Dongxing Wu, Fumihiko Kawamori, Asaka Ikegaya, Takuya Watanabe, Kazuhito Saitoh, Daisuke Takechi, Yoichi Murakami, Daisuke Shichi, Katsumi Aso, Shuji Ando

**Affiliations:** University of Shizuoka and Global Center of Excellence Program, Shizuoka City, Japan (Gaowa, Y. Yoshikawa, N. Ohashi, D. Wu,);; Shizuoka Institute of Environment and Hygiene, Shizuoka City (F. Kawamori, A. Ikegaya);; Seirei Hamamatsu General Hospital, Shizuoka (T. Watanabe, K. Saitoh, D. Takechi);; Seirei Mikatagahara General Hospital, Shizuoka (Y. Murakami, D. Shichi);; Seirei Numazu Hospital, Shizuoka (K. Aso);; National Institute of Infectious Diseases, Tokyo, Japan (S. Ando)

**Keywords:** Anaplasma phagocytophilum, anaplasmosis, serodiagnosis, p44/msp2, P44 immunodominant protein, Japan, vector-borne infections, bacteria

**To the Editor:** Human granulocytic anaplasmosis (HGA) is an emerging tick-borne infectious disease caused by *Anaplasma phagocytophilum*, an obligatory intracellular bacterium (*1*). Recently, 2 cases of HGA were identified by a retrospective study in Japan (*2*). For serodiagnosis of HGA, *A. phagocytophilum* propagated in HL60 cells is usually used as an antigen, especially by indirect immunofluorescent assay (IFA) (*3*). However, the serum from these 2 patients in Japan reacted with antigens of *A. phagocytophilum* cultured in THP-1 cells rather than in HL60 cells in IFA (*2*). In *A. phagocytophilum*, a *p44/msp2* multigene family encoding multiple 44-kDa immunodominant major outer membrane protein species (so-called P44) exists on the genome, and these multigenes are similar, but not identical, to each other, and the bacterium generates antigenic variations because of gene conversion (*4*). The previous studies showed that *A. phagocytophilum* expresses predominantly 2 species of *p44*/*msp2* transcripts in THP-1 cells, but it produces the variation of P44 protein species in HL60 cells (*2*,*5*). This finding strongly suggested that *A. phagocytophilum* grown in THP-1 cells differs serologically from that in HL60 cells. Our serologic analysis found 4 recent cases of HGA in Japan by using infected THP-1 and HL60 cells as antigens, and some P44 immunoreactive protein species of *A. phagocytophilum* that were associated with the respective cell line cultures, binding to antibodies from the 4 patients’ serum, also were identified.

In 2010 and 2011, nine patients in Shizuoka Prefecture, Japan, who had rickettsiosis-like symptoms, were suspected to have Japanese spotted fever or scrub typhus, but they were serologically negative by IFA. Therefore, IFA for HGA was conducted. In 4 of the patients, antibodies to *A. phagocytophilum* were detected in serum by using *A. phagocytophilum* cultured in THP-1 and HL60 cells as antigens (Table). In IFA tests for HGA, IgM and/or IgG from the patients’ serum samples reacted with *A. phagocytophilum* cultured in THP-1, HL60, or both, and the seroconversions were observed in convalescent-phase serum from all patients. The clinical manifestation and laboratory findings for the 4 patients are summarized in the Technical Appendix Table). Western blot analysis further confirmed the specific reaction to P44 protein antigens (P44s) of *A. phagocytophilum* cultured in THP-1 and HL60 and to recombinant P44–1 protein (rP44–1) in the serum samples (Technical Appendix Figures 1 and 2), supporting the IFA results in the Table.

**Table:** Immunofluorescence antibody titers to *Anaplasma phagoctophilum* in serum from 4 patients with human granulocytic anaplasmosis and reactive rP44 protein species, Japan, 2010–2011*

Patient no.	Time after illness onset, d	Antigen				
		*A. phagocytophilum* propagated in THP-1 cells (rP44 species)			*A. phagocytophilum* propagated in HL60 cells (rP44 species)	
		IgM	IgG		IgM	IgG
1	1	80 (r60)	<20		80 (r18ES)	<20
	15	160 (r60)	<20		160 (r18ES)	<20
	30	320 (r60)	20 (r60)		320 (r18ES)	<20
2	13	40	40 (r47E)		<20	20
3	3	40	80 (r60)		<20	20 (r18ES)
	7	40	80 (r60)		<20	20 (r18ES)
	24	80 (r60)	160 (r60)		<20	40 (r18ES)
4	4	160 (r47E)	40		<20	<20
	15	160 (r47E)	80		<20	<20

*Three recombinant P44 (rP44) protein species (r18ES, r47E, r60) were prepared and either one bound to antibodies in each serum sample from 4 patients in Western blot analyses (online Technical Appendix Figure 4, wwwnc.cdc.gov/EID/article/20/3/13-1337-Techapp1.pdf). r18ES represents rP44–18ES immunoreactive outer membrane protein that is known to predominate in *A. phagocytophilum* cultured in HL60 cells (*6*). r47E and r60 show rP44–47E and rP44–60 proteins, respectively, that are dominantly transcribed in *A. phagocytophilum *propagated in THP-1 cells (*2*).

To identify P44 immunodominant protein species binding to antibodies from the patients’ serum, we selected P44–47E and P44–60 proteins that are dominantly expressed by *A. phagocytophilum* propagated in THP-1 cells (*2*) and P44–18ES protein that frequently predominates by *A. phagocytophilum* cultured in HL60 cells (*6*) as representatives for the preparation of recombinant proteins. The central hypervariable regions of the respective P44 proteins (Technical Appendix Figure 3) were produced as recombinant proteins in vitro by insect cell–free protein synthesis system (Transdirect Insect Cell Kit; Shimadzu Co., Kyoto, Japan) (*7*) to avoid the strong nonspecific reaction with human serum that occurs in the *Escherichia coli* expression system. In Western blot analyses using these 3 recombinant P44 proteins (rP44–60 and rP44–47E for THP-1 and rP44–18ES for HL60) as antigens, most of the serum from the patients was reactive with *A. phagocytophilum* cultured in THP-1 cells in IFA bound to either rP44–60 or rP44–47E, whereas the patients’ serum reactive with *A. phagocytophilum* cultured in HL60 cells in IFA bound to rP44–18ES (Technical Appendix Figure 4; Table). This finding strongly supports the results of IFA and Western blot analyses with the infected THP-1 and HL60 cells.

In Japan, rickettsioses such as Japanese spotted fever and scrub typhus, caused by *Rickettsia japonica* and *Orientia tsutsugamushi*, respectively, occur frequently. However, fever of unknown cause and rickettsiosis-like symptoms still occur in some patients. Detection of *A. phagocytophilum* in ticks was first reported in 2005 in central Japan (*8*). Since then, DNA of *A. phagocytophilum* has been detected in ticks inhabiting several places of Japan (*9*,*10*). However, little was known about human infection with *A. phagocytophilum* for many years, probably because of the poor selection of the culture cell line used as infected cell antigens for serodiagnosis. Our previous study first documented HGA in Japan and recommended that *A. phagocytophilum* propagated in THP-1 and in HL60 cells be used as antigens to avoid misdiagnosing cases of HGA. Our current study demonstrates the presence of specific antibodies against the central hypervariable regions of P44–47E, P44–60, or P44–18ES proteins that predominate in infected THP-1 or HL60 cells, probably being suitable as protein antigens for serodiagnosis of HGA. The rP44–1 protein whose recombinant plasmid had previously been constructed for *E. coli* expression system may be available as well. Thus, our study provides substantial information about the usefulness of suitable P44 immunoreactive protein species of *A. phagocytophilum* as antigens for serodiagnosis of HGA.

Technical Appendix Clinical manifestations and laboratory findings of 4 patients with human granulocytic anaplasmosis, Japan, 2010–2011; Western blot analyses of serum from these 4 patients using recombinant P44-1 protein and *A. phagocytophilum*–infected THP-1 and HL60 cells as antigens; amino acid sequence comparison of the hypervariable regions of 3 recombinant P44 protein species; and identification of P44 protein species binding to antibodies.
