# Supplementary material for: Anaplasma phagocytophilum Antibodies in Humans, Japan, 2010–2011
Source: Emerg Infect Dis. 2014 Mar;20(3):508–9. doi: 10.3201/eid2003.131337 (PMC3944852; doi:10.3201/eid2003.131337)
Supplement: Technical Appendix — Clinical manifestations and laboratory findings of 4 patients with human granulocytic anaplasmosis, Japan, 2010–2011; Western blot analyses of serum from these 4 patients using recombinant P44-1 protein and A. phagocytophilum–infected THP-1 and HL60 cells as antigens; amino acid sequence comparison of the hypervariable regions of 3 recombinant P44 protein species; and identification of P44 protein species binding to antibodies. [file 13-1337-Techapp-s1.pdf]

Article DOI: http://dx.doi.org/10.3201/eid2003.131337

Anaplasma phagocytophilum Antibodies in Humans, Japan, 2010–2011

Technical Appendix

Technical Appendix Table. Clinical manifestations and laboratory findings of 4 patients with HGA, Japan, 2010–2011*

Patient	Age, y/sex	Occupation	Hospital	Days with fever after possible tick bite	Symptom onset	Fever	Rash	Additional symptom/sign	Underlying disorder	Laboratory findings (reference values)†	Treatment
1	87/F	Farmer	A	5 d after working on farm	2010 May	39.7°C	Yes	Malaise	Hypertension, hyperlipidemia	WBC, 8.7 × 10^9 cells/L (3.5–9.2 × 10^9 cells/L); Plt, 196 × 10^9 cells/L (155–365 × 10^9 cells/L); AST, 145 U/L (<38 U/L); ALT, 95 U/L (<36 U/L); LDH, 318 U/L (125–237 U/L); CRP, 16.6 mg/dL (0.3 mg/dL)	Minocycline (200 mg/d) by oral administration for 24 d
2	49/M	Electronics worker	B	5 d after construction in forest	2010 May	39°C	Yes	Anorexia, diarrhea, acute renal failure	Diabetes	WBC, 13.1 × 10^9 cells/L (3.5–9.2 × 10^9 cells/L); Plt, 190 × 10^9 cells/L (155–365 × 10^9 cells/L); AST, 69 U/L (<38 U/L); ALT, 49	Minocycline (200 mg/d) by oral administration for 21 d

Patient	Age, y/sex	Occupation	Hospital	Days with fever after possible tick bite	Symptom onset	Fever	Rash	Additional symptom/sign	Underlying disorder	Laboratory findings (reference values)†	Treatment
										U/L (<36 U/L); LDH, 236 U/L (125–237 U/L); CRP, 6.6 mg/dL (0.3 mg/dL); BUN, 54 mg/dL (9–21 mg/dL); Cre, 5.62 mg/dL (0.6–1.2 mg/dL)	
3	83/M	Retired	A	3 d after traveling to mountains	2011 Jun	38.2°C	No	None	Diabetes, hypertension, arteriosclerosis obliterans	WBC, 6.3 × 10^9 cells/L (3.5–9.2 × 10^9 cells/L); Plt, 88 × 10^9 cells/L (155–365 × 10^9 cells/L); AST, 34 U/L (<38 U/L); ALT, 27 U/L (<36 U/L); LDH, 266 U/L (125–237 U/L); CRP, 10.9 mg/dL (0.3 mg/dL)	Minocycline (200 mg/d) by intravenous administration for 6 d and then oral administration for 9 d
4	38/M	Industry worker	C	18 d after working in depository of industry	2011 Jun	39°C	Yes	Chills, arthralgia, headache	None	WBC, 2.1 × 10^9 cells/L (3.5–9.2 × 10^9 cells/L); Plt, 126 × 10^9 cells/L (155–365 × 10^9 cells/L); AST, 31 U/L (<38 U/L); ALT, 53 U/L (<36 U/L); LDH, 277 U/L (125–237 U/L); CRP, 0.7 mg/dL (0.3 mg/dL)	Minocycline (200 mg/d) by oral administration for 10 d

Patient	Age, y/sex	Occupation	Hospital	Days with fever after possible tick bite	Symptom onset	Fever	Rash	Additional symptom/sign	Underlying disorder	Laboratory findings (reference values)†	Treatment

*Three hospitals were located in Shizuoka Prefecture, Japan. Three patients excluding case-patient 4 were hospitalized for treatment of HGA. HGA, human granulocytic anaplasmosis; WBC, white blood cells (leukocytes); Plt, platelets (thrombocytes); AST, aspartate aminotransferase; ALT, alanine aminotransferase; LDH, lactate dehydrogenase; CRP, C-reactive protein; BUN, blood urea nitrogen; Cre, creatinine.

†Laboratory value at time of hospital admission.

Technical Appendix Figure 1. Western blot analyses of serum from 4 case-patients, performed by using recombinant P44-1 protein (rP44-1) and *Anaplasma phagocytophilum*–infected THP-1 cells as antigens, Japan, 2010–2011. The recombinant *Escherichia coli*–producing rP44-1 was kindly provided by Yasuko Rikihisa at The Ohio State University (Columbus, OH, USA). The preparation of purified rP44-1 protein and the rabbit hyperimmune serum (positive serum control) has been described (*2*). A human serum sample (negative control) is shown. The primary human serum samples tested were 250-fold diluted, and the rabbit serum as positive control was 10,000-fold diluted. The goat antihuman IgG and IgM alkaline phosphatase conjugates (Life Technologies, Grand Island, NY, USA) were used as secondary antibodies. rP, recombinant P44-1 protein (rP44-1); In; infected THP-1; Un, uninfected THP-1; M, marker in size.

Technical Appendix Figure 2. Western blot analyses of serum from 4 case-patients, performed by using recombinant P44-1 protein (rP44-1) and *Anaplasma phagocytophilum*–infected HL60cells as antigens, Japan, 2010–2011. The rabbit hyperimmune anti-rP44-1 serum and a human serum sample were used as positive and negative controls, respectively. rP, recombinant P44-1 protein (rP44-1); In, infected HL60; Un, uninfected HL60.

Technical Appendix Figure 3. The amino acid sequence comparison of the hypervariable regions of 3 recombinant P44 protein species (rP44–47E, rP44–60, and rP44-18ES). The rP44–47E protein encodes 651 bp in the 460–1,110-bp position of open reading frame corresponding to 217 amino acids. The rP44-60 protein encodes 633 bp in the 40–672-bp position of the truncated P44-60 corresponding to 211 amino acids. Both P44–47E and P44–60 are dominantly transcribed in *Anaplasma phagocytophilum* cultured in THP-1 cells (*2*). The rP44-18ES protein encodes 624 bp in the 460–1,083-bp position of P44-18E open reading frame corresponding to 208 aa. The P44-18ES protein is known to predominate in *A. phagocytophilum* propagated in HL60 cells (*6*). The hypervariable regions of 3 recombinant P44 protein species produced have 74.9% identity between rP44–47E and rP44-60, 70.2% between rP44–47E and rP44–18ES, and 70.2% between rP44–60 and rP44–18ES. For

preparation of these 3 rP44 protein species (rP44–47E, rP44–60, and rP44–18ES), the DNA including a central hypervariable region of each P44 protein species was artificially synthesized in consideration of codon use for insect. After synthesis, the DNA region was cloned into a pUC57 plasmid. Then, the insert region with in-fusion tags in both 5′ and 3′ ends was amplified, and the PCR product was cloned into pTD1 expression vector by using in-fusion cloning kit (Life Technologies, Grand Island, NY, USA). The mRNA of respective rP44 protein species was transcribed in vitro from the constructed plasmids by T7 RiboMAX express large-scale RNA production system (Promega Co, Madison, WI, USA). Then, the respective recombinant proteins were produced from the transcribed mRNA in vitro by insect cell-free protein synthesis system (Transdirect Insect Cell Kit; Shimadzu Co., Kyoto, Japan) (*7*) and used as antigens for Western blot analysis (see Technical Appendix Figure 4).

Technical Appendix Figure 4. Western blot identification of P44 protein species (r18ES, r47E, or r60) binding to antibodies in 4 case-patients. r18ES represents rP44-18ES protein antigen that is known to predominate in *Anaplasma phagocytophilum* cultured in HL60 cells (*6*). r47E and r60 show rP44–47E and rP44–60 proteins, respectively, that are dominantly transcribed in *A. phagocytophilum* propagated in THP-1 cells (*2*). The hypervariable regions of 3 recombinant proteins with 23–25 kDa (r47E, r60, and r18ES, see Technical Appendix Figure 3) were all detectable by the rabbit hyperimmune anti-rP44-1 serum as a positive serum control previously prepared (2) as shown in the top left panel.
